# Lutein Production and Extraction from Microalgae: Recent Insights and Bioactive Potential

**DOI:** 10.3390/ijms25052892

**Published:** 2024-03-01

**Authors:** Eleonora Montuori, Serena Lima, Arima Marchese, Francesca Scargiali, Chiara Lauritano

**Affiliations:** 1 Department of Chemical, Biological, Pharmaceutical and Environmental Sciences, University of Messina, Viale F. Stagno d’Alcontres 31, 98166 Messina, Italy; eleonora.montuori@studenti.unime.it; 2 Department of Ecosustainable Marine Biotechnology, Stazione Zoologica Anton Dohrn, Via Acton 55, 80133 Napoli, Italy; 3 Department of Engineering, University of Palermo, Viale delle Scienze ed. 6, 90128 Palermo, Italy; serena.lima@unipa.it (S.L.); arima.marchese@community.unipa.it (A.M.); francesca.scargiali@unipa.it (F.S.)

**Keywords:** lutein, health effects, antioxidant activity, microalgae, bioactivity, lutein production

## Abstract

Microalgae have been reported to be excellent producers of bioactive molecules. Lutein is a pigment reported to have various beneficial effects for humans, and especially for eye well-being. In the current review, we summarize various methods that have been developed to optimize its extraction and bioactivities reported for human health. Several protective effects have been reported for lutein, including antioxidant, anticancer, anti-inflammatory, and cardioprotective activity. This review also reports attempts to increase lutein production by microalgae by changing culturing parameters or by using pilot-scale systems. Genetic engineering lutein production is also discussed. Considering the increasing aging of the worldwide population will create an increased need for lutein, a viable economic and eco-sustainable method to produce lutein is needed to face this market demand.

## 1. Introduction

Marine organisms are considered a great source of many essential nutrients for human health, and, among these, one of the most studied is the carotenoid lutein. It is a yellow-orange pigment with known protective activities and beneficial effects for humans, including decreasing the risk of cancer, improving cardiovascular health, and benefitting human eye well-being [1]. For instance, its presence in the retina of the human eye has been reported, where it plays a protective role against the damage caused by free radicals and harmful blue light. For this purpose, it is essential to take the right amount of lutein through one’s diet. 

**Figure 1:**
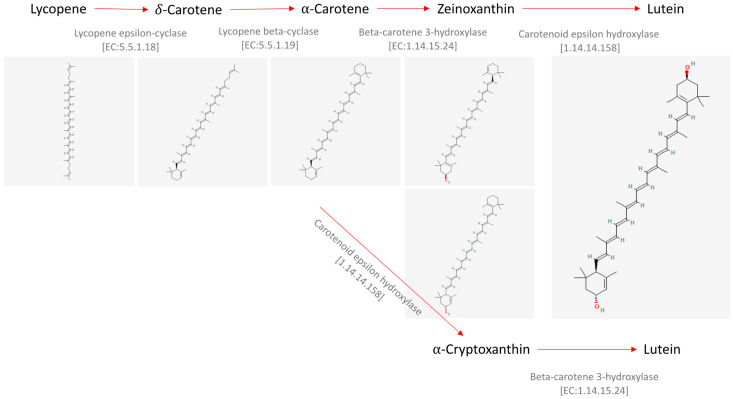
Synthesis of the carotenoid lutein starting from lycopene. Chemical structures were retrieved from the public database PubChem [4,5,6,7,8,9].

Carotenoids are molecules known to have antioxidant properties due to their rich composition in double bonds, which are able to react with reactive oxygen species with radical scavenging properties. Lutein is a compound with the formula C_40_H_56_O_2_, and its secondary structure is as reported in Figure 1. It is a xanthophyll, classified as a carotenoid. Xanthophylls are photosynthetic light-harvesting compounds known to be involved in photoprotection, in the presence of excessive light, by dissipating energy, but they are also structural entities within the light-harvesting complex (LHC).

Lutein is known to be synthesized by plants as a defensive molecule against ultraviolet light, pathogens, or predators [2]. It is not synthetized by animals but is accumulated after ingestion and gives rise to the yellow color, for instance, of egg yolk, animal fat, and human eye retinal macula [3]. Regarding its biosynthetic route, it is known to be synthetized from lycopene and α-carotene, as shown in the Kyoto Encyclopedia of Genes and Genomes (KEGG) PATHWAY Database (https://www.kegg.jp/pathway/map00906+C08601; accessed on 9 January 2024) and briefly shown in Figure 1.

Lutein, in its fatty acid ester form, is extensively found in flowers, yellow autumn leaves, fruits, and vegetables. Metabolites of lutein, including various geometrical isomers, dehydration, and oxidation products, have been identified in human plasma extracts. Lutein can exist in eight stereoisomeric forms due to the presence of three stereocenters at the 3, 3′, and 6′ carbon atoms [10]. 

Today, commercial production of lutein mainly relies on the bright yellow petals of the marigold flowers of the genus *Tagetes*. This source has several disadvantages depending on seasonality, and results are technically relatively complex [11]. Microalgae produce lutein at a rate three to six times higher compared to marigold flowers and require less water [12]. Studies have reported that chemical synthesis allows one to obtain only very low lutein yields [13], and it has been suggested to explore new lutein sources, such as microalgae. Lutein quantities reported for various microalgae will be discussed in the paragraphs below.

Marine organisms, such as microalgae and fish (e.g., salmons and sardines) are a rich source of lutein [14]. Saha et al. [11] reported that from some species of freshwater and marine microalgae, it is possible to obtain 5 g of free lutein per kg of algal biomass [15]. Fish feeding on microalgae accumulate it in their tissues and are therefore rich in lutein. A rich diet based on these fish can be an excellent source for lutein uptake, helping to maintain healthy vision even in old age and to prevent degenerative eye diseases and other pathologies, as explained in the paragraphs below. In fact, many studies confirm the importance of lutein for the prevention of human diseases [16,17,18].

The lutein obtained from these organisms can be used for the formulation of food supplements and also as food colorant (E161b) approved by the European Union [19]. In particular, microalgae are the principal source of lutein from the marine environment. Some freshwater species of microalgae are studied for their production of large amounts of lutein, such as *Chlorella zofingiensis* [20] and *Auxenochlorella protothecoides* [21], but other Chlorophyta, such as *Muriellopsis* sp. [22,23] and *Scenedesmus* sp. [24], have also been reported as great sources of lutein. In fact, these microalgae are used in the form of food supplements [19,25]. There are, for instance, various products based on *Chlorella* sp., which are available on the online shopping market in the form of tablets or powder and produced by companies based all over the world.

The lutein market is mainly related to dietary supplements and functional foods. Considering the increasing ageing of the worldwide population (and, hence, an increase in eye-related disorders), the consciousness of the beneficial roles of lutein in human health, and also its use in foods and beverages, lutein use has increased. While the global market for this compound was USD 371 million in 2023, according to MARKETSANDMARKETS [26], this is projected to reach USD 488 million by 2028, with a CAGR of 5.6%.

## 2. Extraction Procedures

**Figure 2:**
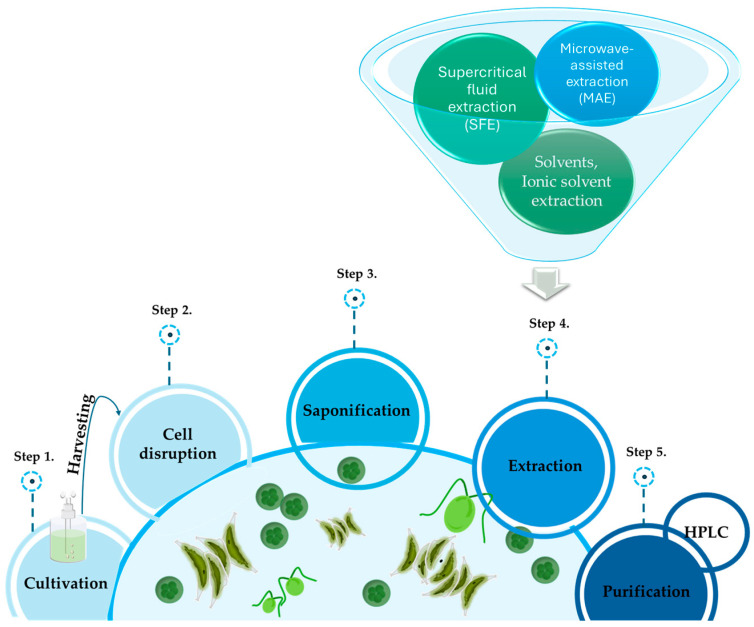
The figure graphically summarizes the various steps commonly followed for lutein extraction.

Marine microalgae are organisms rich in lutein, and over the years, various methods have been developed to optimize its extraction (Figure 2) [11]. There are several lutein extraction methods for marine organisms, including solvent extractions, extraction with supercritical CO_2_, extraction with ionic solvents, microwave-assisted extraction (MAE), and extraction with ultrasounds [27]. These methods are explained in the following paragraphs. 

### 2.1. Solvent Extraction

Extraction with organic solvents is the most commonly used method to obtain pigments from biomasses. Usually, non-polar solvents are used to extract carotenoids, given their high hydrophobicity, such as n-hexane, dichloromethane, dimethyl ether, and methanol, along with other solvents such as acetone and octane [28]. The biomass is macerated for a defined time in the chosen solvent and then homogenized to conduct a phase separation so as to separate the phase with the compound of interest. For lutein extraction, the most used solvents are acetone and ethanol, but also dichloromethane and methanol [29], although they have a much wider environmental impact, being highly toxic. The biomass mixture with the solvent is filtered, and the solvent is evaporated to obtain pure lutein. Following the extraction of biomass by the conventional method for obtaining pigments [30], the extract needs a further purification step to separate the carotenoids from the mixture and obtain high-purity lutein in order to meet marketing requirements [11,31,32]. To meet these needs, in 2002, Li and collaborators [31] set a simple and effective method to optimize the production of high-purity lutein following saponification and crude extraction with dichloromethane from the microalga *Chlorella vulgaris*. The raw extract obtained contained 30% of lutein. Since the raw extract contained water-soluble and liposoluble impurities, Li et al. measured the breakdown values of lutein in a two-phase ethanol–water-dichloromethane system in order to choose the best washing conditions to remove impurities. Dichloromethane extract was washed with 30% aqueous ethanol until the aqueous phase was almost colorless and the pH was almost neutral. The organic phase was then dried with rotary vaporization at 40 °C. Lutein obtained in this phase contained 70% lutein (*w*/*w*). Later, lutein was dissolved in 85% aqueous ethanol, and the fat-soluble impurities were extracted twice with hexane. The lutein obtained after filtration was dry-fed, and its purity determined with HPLC. The final purity was between 90 and 98%, and the yield was between 85 and 91%. In conclusion, the authors found that extraction with hexane was the best to remove the liposoluble impurities [31]. A few years later, Chan and collaborators [33] carried out a study to improve lutein’s commercial viability. They have chosen as their source of lutein the microalga *Scenedesmus obliquus* CNW-N as it has been reported to have a content of more than 0.25% of lutein. They compared the conventional method based on solvent extraction with their modified method to increase lutein yield from microalgal biomass. In the conventional method, the total extraction of pigments (chlorophyll and carotenoids) is obtained. The modified method instead allowed one to obtain only the carotenoids. The steps preceding the purification of lutein in the modified method are the cell-disrupting step [34], saponification, and solvent extraction. The cell-disrupting step can be achieved through several methods, such as by using an ultrasonicator or a bead-beater. For each method, 10–15 mg of algal dry cell weight was used. Among all methods, the disruption with bead-beater was the one that provided the best disruption results. The second step is saponification, which is essential to remove the ionizable lipids, completely disrupt the cells, and convert the esterified lutein contained in the microalgae into its free form. The microalgal biomass was mixed with potassium hydroxide KOH solution at a concentration of 0–80% (*w*/*v*). Chan et al. [33] modified the conventional saponification step to reduce the overall extraction time by 24 h (from 30 h to 6 h). The last step is solvent extraction. Chan et al. selected seven organic solvents: acetone, petroleum ether, n-hexane, diethyl ether, chloroform, dichloromethane, and methanol. The ratio of organic solvents to the sample mixture was 2:1 (*v*/*v*). The diethyl ether was found to be the best among solvents to extract lutein and was used to identify the optimal solvent-to-raffinate (S/R) ratio, leading to the highest lutein content extracted. The lutein content was measured by purging the organic solvent with an N_2_ flow and then dissolving the precipitate with acetone for HPLC measurements. Lutein obtained by the conventional method was 10–20% less than that obtained by the modified method. Recently, extraction methods have been developed with the use of green solvents, such as ethanol and biphasic water solvent mixtures [35]. It is interesting to note that lutein is marketed in the form of oil extracts ranging from 5 to 60%, since its crystalline form is unstable. Therefore, the algal biomass can be extracted with olio-resin in order to obtain 25% lutein extracts that are already marketable [36]. Various studies on methods for the extraction of lutein from microalgae were also included in the review by Saha et al. [11].

Kanda et al. yielded 0.30 mg g^−1^ dry lutein from wet microalga *Monostroma nitidum* using a method employing dimethyl ether (DME). DME resulted an ecofriendly and safe method to obtain a high yield of lutein with unheated drying of wet macroalgae in a single step. As the consumption of liquefied DME increased, the amount of water in the biomass was reduced. Almost all of the water was removed when 216 g DME was reached. In this way, there was no need for any other drying step. The liquefied DME was able to break the hydrogen bonds that were created between the water and the cells of *M. nitidum*. The water was immediately mixed with liquefied DME during the first stage of extraction. The water was extracted with a delay due to the resistance to transport in the latter phase of extraction. This transport resistance can be due both to spatial resistance, such as movement in a narrow space, and to physical-chemical resistance, such as the strong hydrogen bond interactions between cellulose, polysaccharides, and water [37]. 

Recently, in 2022, Patel et al. [29] evaluated what might be the best condition for extracting the maximum amount of lutein from microalgae. They cultivated *Chlorella sorokiniana* Kh12 under mixotrophy. The condition of mixotrophy 2X-(HT)-9k produced maximum biomass (3.46 g L^−1^) and lutein equal to 13.69 mg/g, which is among the highest yields reported so far. Seven minutes of bead beating was found to be the best disruption method to break the walls of the algae (compared to French press, mortar and pestle, and ultrasonic bath), obtaining maximum lutein (7.56 mg g^−1^) extraction, while methanol was found to be the best solvent to extract lutein from *C. sorokiniana* [29].

### 2.2. Supercritical Fluid Extraction (SFE)

SFE is a very efficient technique to extract natural compounds. With this method, lutein is effectively extracted using CO_2_ and ethanol as cosolvent. CO_2_, being chemically inert, non-flammable, non-toxic, retained from degradation [38], and especially economical, is the supercritical solvent preferred in SFE techniques [19,39]. The extraction process takes place in an extraction chamber, where the CO_2_ is brought to a supercritical state by applying pressure and temperature. In this state, CO_2_ has the properties of a gas and a liquid simultaneously, making it a very efficient solvent for the extraction of compounds such as lutein. During extraction, supercritical CO_2_ is passed through the plant material containing lutein. Supercritical CO_2_ acts as a solvent, dissolving lutein and other compounds. Then, the CO_2_ is separated from the solution containing lutein, allowing for purification. The advantage of using CO_2_ as a solvent is that it can be easily removed from the solution, leaving the purified lutein without solvent residues. This extraction method is widely used in the food and pharmaceutical industry to obtain lutein from natural sources. Supercritical CO_2_ extraction is a sustainable method because CO_2_ can be recovered and recycled, thus reducing environmental impacts. In addition, the use of low pressure during the extraction process can contribute to greater sustainability. Interest has been growing in this approach to recovering pharmaceutical and nutraceutical compounds precisely because it guarantees clean extracts and is not toxic. It would seem that, coupled with a co-solvent, the SFE method is among the most promising approaches for lutein extraction. Miguel et al., in their work in 2008, conducted a solvent extraction of carotenoids, following which they mixed the carotenoid-containing solvent with supercritical CO_2_ and optimized the pressure and temperature conditions to promote lutein precipitation [40]. According to Yen et al., the addition of polar co-solvents such as methanol or ethanol may increase lutein extraction efficiency [41]. They focused their research on the use of SFE methods for lutein extraction from the microalga *Scenedesmus* sp. Optimal parameters to increase the recovery yield of lutein have been tested, such as a temperature of 70 and pressure of 400 bar, whichh led to a recovery of 76.7% of lutein. There are still some criticisms in the development of techniques that use supercritical CO_2_, as shown by Ruen-Ngam et al., which assessed the impact of the CO_2_ flow rate for lutein extraction. They showed that the recovery of lutein (equal to 53%) was higher at a 0.4 mL min^−1^ flow rate, but with an increase of the flow rate to 0.5 mL min^−1^, the recovery of lutein was reduced. This phenomenon may be due to the prevalence of resistance to interparticulate diffusion [42].

A study by Gilbert-López et al. in 2017 [43] also aimed to develop a downstream platform for the exploitation of microalgal biomass. The basic approach was to extract and fractionate bioactive compounds from the microalgae Scenedesmus obliquus by applying a sequential process to produce increases in solvent polarity using non-toxic solvents. The phases used were supercritical CO_2_ extraction ScCO_2_, gas-expanded liquids (GXL) using 75% ethanol and 25% ScCO_2_ (*v*/*v*), and pressurized liquid extraction (PLE) using water. Lutein and β-carotene were the main pigments identified in the extracts by HPLC coupled to diode matrix detectors and mass spectrometry (HPLC-DAD-MS/MS), which were preferably extracted in the GXL phase [43].

### 2.3. Ionic Solvent Extraction

Another effective method to obtain lutein from marine organisms is the use of ionic solvents, ion-based liquids. Ionic solvents can be designed to be selective for lutein and can be used to extract lutein efficiently. These solvents are maintained in a liquid state at moderate temperatures from 0 °C to 140 °C [35,44]. Ionic solvent extraction draws attention because it combines both cellular disruption and extraction steps. Among the used solvents, there was the dipropylammonium dipropylcarbamate-methanol (DPCARB) [45,46]. The use of DPCARB has been reported to reduce the extraction time to 30 min compared to conventional methods with solvents by increasing the yield of lutein obtained to 0.96% DW (dry weight) [46]. Natural deep eutectic solvents (NADES), considered a subclass of ionic liquid solvents [47], are solvents that meet the criteria of green chemistry as they are biodegradable and economical [48]. These solvents were used for the recovery phase of lutein during purification, obtaining 4.41 mg g^−1^ against 3.91 mg g^−1^ lutein recovered with conventional solvents [49].

### 2.4. Microwave-Assisted Extraction (MAE)

Microwave-assisted extraction (MAE) offers a faster extraction process compared to conventional methods [50,51]. With this technique, microwaves are used to heat the samples in order to facilitate the release of bioactive compounds. It provides uniform heating throughout the sample, ensuring efficient extraction of the target compound. Another important aspect is that MAE requires less energy compared to traditional extraction methods, making it a more environmentally friendly option [48,50,52]. 

Low et al., in 2020 [53], described an optimization of a novel protocol based on the microwave-assisted binary phase solvent extraction method (MABS) for the extraction of lutein from *Scenedesmus* sp. With this protocol, 11.92 mg/g of lutein was recovered from the biomass. They found that the best binary phase solvent composition was a 60% potassium hydroxide solution with acetone in the ratio of 0.1 (mL mL^−1^). The conditions that allowed them to obtain the maximum content of lutein were 55 °C treatment temperature, 36 min extraction time, 0.7 (mg mL^−1^) biomass–solvent ratio, 250 W microwave power, and 250 rpm stirring speed [53].

Very recently, in 2022, Leema et al. [52] purified and characterized lutein from the microalga *Chlorella sorokiniana* using the optimized microwave-assisted extraction (MAE) method. The optimized conditions for microwave-assisted alkali pre-treatment resulted in a 3.26-fold increase in lutein yield compared to conventional extraction. The effectiveness of the MAE process was confirmed through visualization of the extracted algal biomass using scanning electron microscopy and X-ray diffraction analysis, which showed a significantly higher crystallinity index in the treated biomass. The lutein yield using the optimized MAE conditions was reported to be 20.69 ± 1.2 mg g^−1^. The conventional extraction method yielded lutein at a lower rate of 6.35 ± 0.44 mg g^−1^. X-ray diffraction analysis showed a significantly higher crystallinity index in the microwave-assisted alkali-treated biomass (83.85%) compared to the untreated control (17.28%) [52].

### 2.5. Ultrasonic Extraction

Ultrasonic extraction is an approach that uses high-frequency sound waves to break the cell walls of microalgae to release lutein. The organisms are immersed in a solvent and subjected to ultrasound for a certain period of time. In 2018, Gayathri et al. [54] focused their studies on the extraction of lutein from the marine microalga *Chlorella salina* by using an ultrasound-assisted microextraction (US-ME) technique. They found that the optimal conditions for lutein extraction from *C. salina* were a temperature of 40 °C, extraction time of 30 min, and a frequency of 35 kHz. Under these optimal conditions, the concentration of lutein obtained from *C. salina* was 2.92 ± 0.40 mg g^−1^ DW (dry weight). The results demonstrated that ultrasound-assisted microextraction (US-ME) is a very useful method for extracting lutein from microalgae, specifically from *Chlorella salina*. [54]. Ultrasonic extraction can be made more environmentally friendly by using low energy consumption and reducing extraction time. In addition, the use of ultrasounds can reduce the amount of solvent required for the extraction, contributing to greater sustainability.

### 2.6. Purification of Lutein

The essential step to obtain high-purity lutein is the purification that follows the extraction with the methods mentioned above. Among the mentioned techniques, the most promising method for extracting lutein on a large industrial scale is SFE, although other further studies are necessary to demonstrate the high yield of pure lutein. As mentioned above, the current methods of purification of carotenoids are based on the Willstätter method [19,48]. This name derives from the name of the Nobel Prize winner Richard Willstätter [55], who pioneered methods for the study and purification of secondary metabolites from natural sources, specifically pigments. In various laboratory studies following these extraction steps, the purification is conducted essentially with chromatography techniques, such as counter-current chromatography or solid support chromatography. High-performance counter-current chromatography has been shown to obtain a lutein purity equal to 92% [19,33].

## 3. Lutein: A Versatile Antioxidant in Human Health

Lutein has several effects on human health, depending mostly on its antioxidant capabilities. It has been shown that lutein is a strong antioxidant, with an effect that may vary depending on the cultivar of the plant from which it is extracted [56]. Notably, lutein contained in microalgal extracts presents interesting bioactive properties even without additional purification. For instance, lutein contained in an extract of the microalga *Chlamydomonas reinhardtii* demonstrated antioxidant effects on damaged DNA [57], while lutein contained in an extract of *Tetraselmis* sp. exhibited potent antioxidant, antiviral, and anti-inflammatory properties, suggesting its potential for sustainable industrial applications [58]. 

Mechanistically, in a murine model, lutein was proposed to scavenge reactive species and up-regulated genes associated with antioxidant responses, thereby enhancing oxygen transport [59]. Additionally, studies by Song et al. suggested that lutein, when combined with vitamin C, enhanced the total antioxidant defense system in rats [60]. Human trials further supported the antioxidant effects of lutein. In particular, Jȩdrzejczak-Pospiech et al. showed that the highest total antioxidant status increase was observed after the intake of the lowest lutein dose (8 mg d^−1^) [61]. Research involving lutein supplementation has also been conducted on infants. While administering lutein to pregnant women with gestational diabetes mellitus is linked to a reduction in oxidative stress in newborns at birth, these effects did not persist significantly after 48 h [62]. Similarly, administering 0.5 mg ± 0.02 mg/kg/d of lutein to newborns from 7 days of age was ineffective in enhancing their biological antioxidant capacity [63]. Conversely, recent findings demonstrated that neonatal supplementation with lutein in the early hours of life enhanced biological antioxidant potential and decreased total hydroperoxide levels in supplemented infants compared to untreated ones. This suggested a reduction in free-radical-induced damage at birth due to lutein supplementation [64].

In human health, lutein has two main roles: acting like a filter for high-energetic and damaging blue light at the eye level and acting as antioxidant [65]. The majority of lutein’s health benefits, along with its isomer zeaxanthin, depend on its antioxidant activity, which also contributes to its anti-inflammatory properties. Oxidative stress underlies the pathogenesis of various diseases, including diabetes, neurodegeneration, atherosclerosis, cancer, eye diseases, diabetic retinopathy, osteoporosis, cardiovascular diseases, skin diseases, liver injury, obesity, and colon diseases [66]. Reactive oxygen species (ROS) are produced both internally by mitochondrial respiration and externally by cytotoxic factors. Lutein, with its free-radical-scavenging activity, mitigates oxidative stress and inflammatory responses in different organs. It is notable that lutein’s efficacy in scavenging ROS may vary depending on the specific species. A study indicates that lutein effectively protects human cells against OH• radicals but shows less efficacy against NO_2_• and O_2_• [67]. 

Furthermore, lutein’s antioxidant effect remains largely consistent whether it is in a free or esterified form [68]. However, achieving a bioactive effect depends significantly on lutein’s bioavailability and transport [69], which are influenced by the formulation used. For instance, research by Evans et al. suggested that a starch-based formulation is to be preferred to an alginate-based product [70]. Additionally, the addition of excipients has been observed to enhance lutein’s bioavailability, as noted by Nemli et al. [71]. Moreover, the method of cooking food containing lutein also affects its bioavailability and effectiveness [72,73,74]. It has been shown that one of the effects of cooking may be the epimerization of the lutein, which is caused also by its natural metabolization by the human body. Products of this phenomenon may be 3-epilutein and 3-oxolutein, which also have a putative protective effect [75]. It is worth noting that lutein is subjected to blanching after the extraction, and this should be considered when formulating products that contain carotenoids as ingredients, for example by adding antioxidant agents. Cui et al. investigated the anti-oxidative and anti-inflammatory effects of lutein isomers on a Caco-2 cell model, revealing that also the type of isomer may significantly impact lutein’s health effects [76]. A remarkable human intervention study demonstrated that a single dose of marine *Chlorella vulgaris* increased the plasma concentrations of lutein and other carotenoids, suggesting potential health benefits associated with the use of *Chlorella vulgaris* as a source of carotenoids, polyunsaturated fatty acid (PUFA), and essential trace elements [77]. The effects of lutein on several human diseases are listed below, according to the most recent literature.

### 3.1. Lutein and Zeaxanthin: Guardians of Vision Health

Lutein and zeaxanthin have been reported to play pivotal roles in safeguarding vision by accumulating in two crucial tissues responsible for visual function: the macula lutea and the lens. This accumulation is tissue-specific; no others carotenoids are accumulated in the human body at the same concentrations [65]. The macula lutea, situated under the retina, owes its yellow color to the presence of these pigments. It contains the highest concentration of photoreceptors responsible for central vision. Here, lutein and zeaxanthin shield the tissue from high-energy blue light, which can cause light-induced retinal damage. Additionally, the macula is particularly susceptible to oxidative damage, where lutein also acts as an antioxidant [65]. The literature strongly supports these assertions. For example, there are several reports providing evidence for the effect of lutein on retinal neurodegeneration in diabetes [78].

In vitro studies have further elucidated the protective role of lutein and zeaxanthin. Murthy et al. investigated their dose-response effect on shielding retinal pigment epithelium from oxidative stress, finding that lutein demonstrated a cytoprotective effect at various concentrations, while zeaxanthin did not show similar efficacy [79]. Kamoshita et al. proposed a mechanism for lutein’s protective role, suggesting that it promotes tight junction repair and suppresses inflammation in a model of photo-stressed mice, thereby reducing local oxidative stress through direct scavenging and likely induction of endogenous antioxidant enzymes [80]. Leung et al. explored the impact of lutein, zeaxanthin, and docosahexaenoic acid (DHA) supplementation on retinal pigment epithelial (RPE) cells under oxidative stress, observing their regulation of inflammatory lipid mediators and reduction in non-enzymatic oxidation of omega-6 PUFA [81]. 

Oxidative-stress-induced cellular senescence contributes significantly to the pathogenesis of age-related macular degeneration (AMD). Liu et al. demonstrated lutein’s protective effect on acute retinal pigment epithelial 19 (ARPE-19) cells against oxidative stress damage induced by H_2_O_2_ [82]. Similarly, Chae et al. found that lutein effectively protected ARPE-19 cells from H_2_O_2_-induced effects [83]. Additionally, lutein mitigated UVB-mediated oxidative damage to retinal pigment epithelial cells [84].

Moving to animal models, lutein treatment over three months in ApoE-deficient mice, a model of genetic hypercholesterolemia with retinal alterations, significantly reduced vascular endothelial growth factor (VEGF) levels and matrix metalloproteinase-2 (MMP-2) activity, leading to improved retinal morphological alterations [85]. Padmanabha et al. investigated the effects of lutein and fatty acids in rats on oxidative stress and inflammation in cataracts, finding that lutein administration, particularly with EPA + DHA, reduced markers of oxidative stress [86]. Sahin et al. demonstrated the efficacy of lutein/zeaxanthin treatment on G-protein-coupled receptors in the retinas of rats exposed to intense LED illumination, significantly improving antioxidant capacity [87]. Additionally, lutein protected vestibulocochlear nerve tissue from acrolein-associated oxidative and proinflammatory damage [88]. Zhang et al. observed that lutein promoted the survival of photoreceptors in Pde6b^rd10^ model mice, which are characterized by a degeneration of photoreceptors typical of retinitis pigmentosa, a retinal disease. They observed that lutein at the optimal protective dose of 200 mg kg^−1^ promoted the survival of photoreceptors compared with control, postponing photoreceptor degeneration [89]. However, intratympanic lutein application (1 mg mL^−1^) showed no protective effect against cisplatin-induced ototoxicity in Wistar rats, despite promising in vitro results [90]. Yu et al. showed the effects of lutein on light-induced retinopathy. They observed that lutein and zeaxanthin were effective in decreasing oxidative stress and preserving photoreceptors against light damage [91].

Human studies have yielded mixed results regarding lutein supplementation. While some studies found no statistically significant effects on age-related maculopathy (ARM) and atrophic age-related macular degeneration (AMD) in participants with 6 mg of lutein supplementation [92], others observed slight improvements in retinal function. In fact, a slight but encouraging improvement in retinal function, although not clinically significant, was observed by Berrow et al. [93]. However, supplementation with meso-zeaxanthin, lutein, and zeaxanthin showed benefits in patients with Alzheimer’s disease, resulting in improvements in visual function and macular pigment augmentation [94]. In patients with AMD, supplementation with lutein complex from marigold flower and wolfberry increased serum concentrations of lutein and zeaxanthin, along with antioxidant enzyme activities and macular pigment optical density. Other parameters were also improved, such as ocular comfort index (OCI) and macular pigment optical density (MPOD) [95]. 

In a separate investigation, a high-dose lutein/zeaxanthin supplement was examined for its impact on MPOD and skin carotenoid (SC) levels in healthy individuals. The supplementation of 20 mg/day of lutein, 4 mg/day of zeaxanthin, and other antioxidants (vitamin C, vitamin E, zinc, copper) for 16 weeks led to a notable increase in MPOD [96]. Moreover, a meta-analysis revealed that supplementation with xanthophyll carotenoids significantly raised macular pigment optical density in patients with AMD [97]. Furthermore, a study focusing on patients with chronic central serous chorioretinopathy investigated the effects of lutein supplementation. The group receiving supplementation exhibited significant improvements in mean visual acuity, along with a substantial reduction (28.6%) in mean subfoveal fluid height [98].

### 3.2. Protection of Skin

Similar to its function as a filter of high-energy blue light in plants and in human eyes, evidence suggests that lutein exerts a protective effect, whether ingested or applied topically to the skin, against UV-light exposure.

For instance, Sansone et al. [99] showed that an alcohol/water extract of the microalga *Tetraselmis suecica*, containing lutein, exhibited potent antioxidant and reparative activity in human lung cancer cells (A549). This extract also reduced prostaglandin E2 (PGE2) levels in cells damaged by H_2_O_2_ and demonstrated tissue-repairing effects on reconstructed human epidermal tissue cells (EpiDerm), suggesting a potential cosmeceutical application for this microalgal species [99]. In another randomized, double-blind, placebo-controlled intervention, researchers investigated the effects of dietary lutein supplementation on minimal erythema dose (MED) as an indicator of the skin’s photoprotective potential. Results indicated that MED significantly increased in the group receiving lutein supplementation, indicating enhanced resistance to erythema production following UV radiation. These findings suggest that dietary lutein supplementation can enhance the skin’s ability to protect against UV-induced damage [100].

### 3.3. Neurodegenerative Diseases

In the sphere of neurodegenerative diseases, inflammation plays a pivotal role, and lutein has shown promise in alleviating symptoms associated with various neurodegenerative conditions. 

Liu et al. demonstrated the protective role of lutein against oxidative stress and apoptosis induced by the Aβ25–35 peptide in bEND.3 cells, which are cerebrovascular endothelial cells, a dysfunction that contributes significantly to neurodegeneration [101]. Furthermore, Hu et al. highlighted the protective effects of lutein and DHA on PC12 cells, a model for neurodegenerative disorders, against hydrogen-peroxide-induced oxidative stress, suggesting their potential as potent antioxidants with preventive or palliative effects on age-related neurodegenerative diseases [102]. Similarly, Chen et al. also found that lutein attenuated oxidative damage and apoptosis triggered by methylglyoxal in PC12 cells via the PI3K/Akt signaling pathway [103]. Pap et al. investigated the impact of lutein on antioxidant enzymes, cytokines, and iron metabolism in BV-2 microglia, the primary immune cells in the central nervous system, under stress conditions. They observed that lutein may have a preventive or suppressive effect on ROS-mediated microglia activation by reducing H_2_O_2_-induced ROS levels, modulating iron utilization, and altering the secretion of anti-inflammatory and pro-inflammatory cytokines in BV-2 cells [104]. In a subsequent study, Pap et al. demonstrated that lutein mitigates the effects of glutamate-induced ROS, inflammation, iron metabolism dysregulation, and lipid peroxidation in SH-SY5Y neuroblastoma cells, which serve as a model for neurodegenerative disorders [105]. In animal models, Fernandez et al. illustrated that lutein-loaded nanoparticles offer protection against locomotor damage and neurotoxicity induced by a Parkinson’s disease model in *Drosophila melanogaster* [106]. Oxidative stress is also implicated in the development of Alzheimer’s disease, characterized by memory impairment due to Aβ plaque formation in the brain. Nazari et al. demonstrated the memory-enhancing effects of lutein in a male rat model of Alzheimer’s disease [107]. Additionally, oxidative stress and inflammation are key contributors to diabetic cerebral and neurological dysfunction. Fatani et al. highlighted the role of lutein in combating oxidative injury and inflammation in the cerebral cortex of diabetic animals [108]. Furthermore, lutein has exhibited protective effects against monosodium-iodoacetate-induced (MIA) osteoarthritis in primary chondrocyte cells [109].

When considering research involving human subjects, it has been observed that phospholipid hydroperoxides (PLOOH) are abnormally elevated in the erythrocytes of individuals with dementia, including those diagnosed with Alzheimer’s disease. In a study involving six healthy subjects, the administration of a single capsule containing food-grade lutein (providing 9.67 mg of lutein) daily for four weeks resulted in the incorporation of lutein into human erythrocytes. Over the course of two and four weeks, there was a reduction in erythrocyte PLOOH levels following ingestion. The antioxidative effect of lutein was confirmed on erythrocyte membranes but not in plasma. These findings suggest that lutein may contribute to the prevention of dementia [110]. In another study, the effect of 2 months of *Chlorella* supplementation (8 g *Chlorella*/day/person; equivalent to 22.9 mg lutein/day/person) on PLOOH and carotenoid concentrations in erythrocytes, as well as plasma, was assessed in 12 normal senior subjects. Following one or two months of treatment, erythrocyte and plasma lutein concentrations increased in the *Chlorella* group but not in the placebo group. In the *Chlorella*-supplemented group, erythrocyte PLOOH concentrations after a total of two months of treatment were lower than the concentrations before supplementation. These results suggest that *Chlorella* ingestion improved erythrocyte antioxidant status and lowered PLOOH concentrations, potentially contributing to the maintenance of normal erythrocyte function and the prevention of senile dementia [111]. Furthermore, it has been shown that high serum levels of lycopene and lutein plus zeaxanthin are associated with a lower risk of Alzheimer’s disease mortality in adults [112]. In another study, phospholipid oxidation (particularly the phospholipid biomarker 1-palmitoyl-2- (5′-oxo-valeroyl)-sn-glycero-3-phosphocholine, POVPC) was analyzed as a marker of Alzheimer’s disease. The study revealed that serum POVPC levels are higher in Alzheimer’s disease patients compared to control subjects and are not reduced by carotenoid supplementation. Additionally, serum POVPC levels were found to correlate with cognitive function [113].

### 3.4. Lutein: A Potential Agent against Inflammation and Cancer

The involvement of acute inflammatory response in cancer development is well documented. Presently, there is a continual quest for novel compounds that could potentially prevent and treat both inflammatory diseases and cancer. Regarding chemotherapy drugs, which are known for their aggressive nature, research efforts have been boosted towards exploring alternative therapies, including natural plant-based substances like lutein. Lutein, a bioactive molecule, has been extensively studied for its potential anti-inflammatory and anticancer properties [114]. Notably, its antioxidant properties have been observed, with oxidized lutein showing increased cytotoxicity against human cervical carcinoma cells (HeLa) compared to normal lutein [115,116]. Various cancer types have been treated with lutein in different forms. A summary of lutein’s effects on various cancer types is reported in Table 1 and commented on below.

For instance, in a study on breast cancer cells MCF-7 and MDA-MB-231, lutein extracted from *Spinacia oleracea* was used at different concentrations (1 and 5 µM for MCF-7; 10 and 20 μM for MDA-MB-231) for a duration of 24 h. Lutein inhibited cell viability in a concentration-dependent manner and suppressed reactive oxygen species. It downregulated the expression of Nrf-2 protein and decreased SOD-2, HO-2 proteins, and XBP-1 transcription factor expression. Additionally, it inhibited phosphorylation of the AKT protein and expression of nuclear factor Nf-κB, while increasing caspase-3 activity, leading to apoptosis [117].

In a different in vivo study, lutein from *Strobilanthes crispa* was supplied to mice with doses of 50 mg/kg/day for 30 days. Lutein interacted with the X alpha receptor (RXRα), inhibiting breast cancer [118]. Another study on breast cancer cells MCF-7 and MDA-MB-468 showed that lutein increased the level of intracellular ROS and the pro-apoptotic/anti-apoptotic BAX/Bcl-2 ratio. It activated p53 protein signaling and up-regulated cellular HSP60, inhibiting tumor cell growth [119]. Despite inconsistencies in its molecular mechanisms, especially regarding its effects on reactive oxygen species and caspases, lutein shows promise in breast cancer treatment.

In non-small-cell lung cancer, lutein inhibited cell invasion and regulated signaling molecules involved in apoptosis. In particular, lutein, tested at concentrations of 25 and 50 μM on non-small-cell A549, caused a regulation of PI3K/AKT signaling molecules, inhibition of the anti-apoptotic signaling molecules Bcl2 and Bcl-XL, and an increase in the expression of the pro-apoptotic protein BAX, which activates the executioner caspase 3. This led to apoptosis of A549 cells [120]. No adverse effects were observed, suggesting its potential as an anti-tumor agent.

Another study showed that lutein reduced gene expression associated with cell proliferation in A549 at concentrations of 10, 30, and 50 μM. It modulated apoptotic proteins, indicating its role in regulating apoptosis-related genes. In particular, lutein was found to reduce the gene expression of CAS3, SOX2, NANOG, SLCA11, and ABCB1, while increasing the gene expression of CD44 and CD133. It also promotes the apoptosis induction in cells by decreasing the anti-apoptotic/pro-apoptotic BCL2/BAX protein ratio [121].

In both examined studies, it can be concluded that lutein acts on the gene expression of control proteins that regulate apoptosis, namely BAX, BCL2, and CAS3.

Studies on colon cancer in vivo demonstrated lutein’s chemoprotective effect by modulating proliferative proteins. Using a lutein extract from marigold flowers, Reynoso-Camacho et al. showed that lutein modulates the proliferative activity of K-ras, PKB, and β-catenin proteins, leading to a chemoprotective effect [122].

In various gastric cancer cell lines (AGS, MKN-74, MKN-1, and SNU-668), lutein was tested at different concentrations (5, 10, and 20 µM) for 24 h. Lutein increased ROS levels and activated NADPH oxidase, increasing the translocation of the p47 phox subunit of NADPH oxidase on the cell membrane. Furthermore, it increased NF-κB activation and apoptotic indices, such as Bax, caspase -3 cleavage, and DNA fragmentation, while reducing Bcl-2. It decreased cell viability in a dose-dependent manner [123].

Lutein extracted from carrot was tested on lymphoid leukemia cells (CCRF-CEM, Jurkat, MOLT-3) at concentrations from 0.5 µM to 100 µM for 24 and 48 h. No effects on apoptosis or cell proliferation were observed [124]. Similarly, in melanoma A375 tumor cells, the use of lutein at various concentrations (from 0.5 µM to 100 µM for 24 and 48 h) did not alter cell viability or membrane integrity [125].

With regard to prostate cancer, lutein extracted from marigold at a concentration of 10 µM for 18 h was tested on PC-3 cells. Lutein altered the expression of genes associated with growth and apoptosis [126].

In an in vivo study concerning hepatocellular carcinoma, lutein extracted from marigold reduced liver levels of enzymes such as alanine transaminase, aspartate transaminase, and alkaline phosphatase; reduced the activity of c-glutamyl transpeptidase; and increased glutathione [127].

In a study on cervical carcinoma, lutein from marigold petals used on HeLa cells at a concentration of 10 µm for different durations (24 h and 48 h) inhibited cell proliferation in a dose-dependent manner and increased ROS concentration. Furthermore, it promoted apoptosis by activating Bax and Caspase-3; there was also a significant downregulation of the expression of Bcl-2 (anti-apoptotic) and upregulation of the expression of Bax (pro-apoptotic) [128].

There are some studies in the literature assessing, in particular, the effect of lutein-rich microalgae extracts, which inhibited tumor growth across various cancer types by modulating apoptotic pathways. For example, an ethanolic extract from Chlorella was tested on lung cancer (A549 cells), cervical cancer (Hela cells), breast cancer (MCF7 cells), hepatocellular carcinoma (Huh7 cells), and cholangiocarcinoma (CCA cells; KKU213A cells), at a concentration of 400 μg mL^−1^. Several effects were observed: a reduction in procaspase-3, -8, and -9; an increase in the enzymatic activity of caspase and a reduction in the anti-apoptosis protein Bcl-2 (which induce cell death); and a reduction in the proteins phosphorylate-AKT and phosphorylate-mTOR, which led to inhibition of AKT/mTOR survival signaling. In all types of tumor cells considered, there was growth inhibition [129].

In another study, ethanolic extracts of *Neochloris oleoabundans* grown under different conditions were tested on three colon cancer cell lines, specifically HT-29, SW480, and HGUE-C-1, by using concentrations of 27, 83, or 250 μg mL^−1^ for 24 h. Results showed that most extracts showed dose-dependent anti-proliferative activity on cells, and that the action varied depending on the cell type. The anti-proliferative activity was linked to the concentration of lutein in the extract [130].

In another study, the effect of methanolic extracts of the microalgae *Granulocystopsis* sp. was assessed on various types of tumors, specifically breast cancer (HTB-22), colorectal cancer (HTB-38), prostate cancer (HBT-81), and skin melanoma (HTB-72) [131]. Each cell line reacts differently to the extract, with cell viability depending on the duration of the treatment. Apoptosis was favored in skin melanoma and prostate cancer cells. An increase in the activity of caspases 3 and 7 was observed in all tumor cells.

Numerous epidemiological studies [132,133,134,135,136,137,138,139] have investigated various tumor types including head and neck cancer, bladder cancer, liver cancer, breast cancer, lung cancer, prostate cancer, colon cancer, esophageal cancer, pancreatic cancer, and renal cell carcinoma. These studies are based on the dietary habits and lifestyle of the study participants, with carotenoids typically sourced from fruits and vegetables. In one specific study [134], it was noted that the risk of colorectal cancer is associated with an interactive effect between dietary intake of lutein/zeaxanthin and the DICER1 rs3742330 polymorphism. Across multiple studies, there is evidence of an inverse relationship between total carotenoid intake and cancer risk. Additionally, another study [140] examined the effects of lutein extracted from Medicago Sativa on various cancer types, including liver cancer (HepG2), breast cancer (MCF-7), lung cancer (A549), prostate cancer (PC3), and colon cancer (HCT116). Results indicated that while lutein exhibited activity against breast and liver cancer, it did not demonstrate efficacy against lung, prostate, or colon cancer.

### 3.5. Diverse Protective Effects across Various Diseases

Research has explored the impact of lutein on various other diseases. For instance, oxidative stress is also pivotal in cardiovascular disease, where hyperhomocysteinemia (HHcy)-mediated atherosclerosis (AS) is driven by oxidative stress and inflammation mechanisms. In a murine model, lutein has been shown to significantly intervene and inhibit Hcy-mediated oxidative stress activation while downregulating the expression of inflammation-associated molecules [141].

Li et al. assessed the role of lutein in protecting against osteoporosis in ovariectomized rats by suppressing inflammation and osteoclast-specific marker (NFATc1) expression through Nrf2 activation [142]. The efficacy of plant extracts rich in lutein for ulcerative colitis has also been shown: alcoholic plant extract of *Tagetes erecta* reduced colitis severity by attenuating inflammatory cytokine secretion and improved the endogenous antioxidant defense in 5% dextran sulfate sodium-induced ulcerative colitis in mice [143]. Mammadov demonstrated the effect of lutein on methotrexate-induced pulmonary toxicity in rats, revealing its ability to prevent biochemical and histopathological oxidative damage [144]. Additionally, lutein intake was significantly associated with lower lung function in current smokers [145]. Radhi and Al-Shawi revealed that lutein mitigated irinotecan-induced liver toxicity in rats [146]. In a rat model of polycystic ovary syndrome, Bandariyan et al. demonstrated that lutein improved antioxidant capacity and oocyte and embryo quality [147]. Zhang et al. highlighted lutein’s protective effects against reproductive injury induced by excessive alcohol in male rats through its antioxidant, anti-inflammatory, and anti-apoptotic properties [148]. Fatani et al. found protective effects against diabetes-induced oxidative stress in testicular cells of rats [149]. Moreover, lutein was shown to prevent cardiac and renal injury in streptozotocin-induced hyperglycemic rats, likely by ameliorating glucose intolerance [150]. Lutein was also effective against ischemia/reperfusion injury in rat skeletal muscle, downregulating oxidative stress and inflammatory mechanisms [151]. Furthermore, lutein exhibited beneficial effects on severe traumatic brain injury through anti-inflammatory and antioxidative mechanisms [152]. Human studies have indicated that the intake of β-carotene, lutein/zeaxanthin, and lycopene is associated with a lower risk of metabolic-dysfunction-associated fatty liver disease [153].

**Table 1:** This table reports anticancer bioactivities identified for lutein, active concentrations, mechanisms of action when available, and relative references.

Types of Cancer	Substance	Source	Model	Concentration	Mechanism of Action	Reference
Breast cancer	Lutein	*Spinacia oleracea*	In vitro (cells) MCF-7 and MDA-MB-231 cells	1 and 5 µM for MCF-7 10 and 20 μM for MDA-MB-231 for 24 h	Inhibition of cell viability in a concentration-dependent manner Increase in cell death with higher concentrations of lutein Suppression of reactive oxygen species (ROS) levels Downregulation of Nrf-2 protein expression Decrease in expression of SOD-2, HO-2 proteins, and XBP-1 Inhibition of Akt phosphorylation and NF-κB expression Enhancement of caspase-3 activity, leading to apoptosis	[117]
	Lutein	*Strobilanthes crispus*	In vivo (mouse) 4T1 breast cancer cell line	50 mg/kg/day for 30 days	Activation of retinoid X receptor alpha (RXRa) by lutein Inhibition of breast cancer growth	[118]
	Lutein	-	In vitro MCF-7, MDA-MB-468 cells	2.0 µM for 48 h	Inhibition of tumor cell growth, induction of cell cycle arrest, and cell death Increase in intracellular reactive oxygen species (ROS) levels Enhancement of the BAX/Bcl-2 ratio Activation of p53 signaling and upregulation of HSP60	[119]
Non-small-cell lung cancer (NSCLC)	Lutein	-	In vitro A549 cells	25 and 50 μM (LC_50_ = 50 μM)	Inhibition of cell invasion and migration properties Suppression of PI3K/AKT signaling pathway Inhibition of intrinsic anti-apoptotic signaling molecules Bcl2 and Bcl-XL, leading to apoptosis Increase in expression of pro-apoptotic protein BAX, activating caspase 3 and inducing apoptosis	[120]
	Lutein	-	In vitro A549 cells	10, 30, 50 μM	Decrease in BCL2/BAX protein ratio, promoting apoptosis Reduction in gene expression of CAS3, SOX2, NANOG, SLCA11, and ABCB1 Increase in gene expression of CD44 and CD133	[121]
Colorectal Cancer	Lutein	Marigold flowers (*Tagetes erecta*)	In vivo		Chemoprotective effect against colon cancer Modulation of proliferative activity of K-ras, PKB, and β-catenin proteins	[122]
	Extract of microalgae Pressurized liquid extraction (EtOH)	Microalgae *Neochloris oleoabundans*	In vitro HT-29, SW480 HGUE-C-1 cells	27, 83 or 250 μg mL^−1^ for 24 h	Different extracts exhibit dose-dependent anti-proliferative activities on cells	[130]
Gastric Cancer	Lutein	Cayman Chemical n.10010811	In vitro AGS, MKN-74, MKN-1, and SNU-668 cells	5, 10, and 20 µM for 24 h	Induction of a dose-dependent decrease in cell viability Increase in ROS levels and induction of NADPH oxidase activation, promoting translocation of p47 phox subunit to the cell membrane Enhancement of NF-κB activation and induction of apoptotic indices, including Bax, caspase-3 cleavage, and DNA fragmentation Reduction in Bcl-2 levels	[123]
Lymphoid Leukemia	Lutein	*Daucus carota L*.	In vitro CCRF-CEM, Jurkat, MOLT-3 cells	0.5 µM to100 µM for 24 and 48 h	No effect on apoptosis or cell proliferation	[124]
Melanoma	Lutein	-	In vitro A375 cells	0.1, 0.3, 1, 3 and 10 µM of lutein for 24 h	No alteration in melanoma cell viability or membrane integrity by lutein	[125]
Prostate Cancer	Lutein	Marigold	In vitro PC-3 cells	10 µM for 18 h	Alteration of expression of biomarker genes associated with growth and apoptosis by lutein	[126]
Hepatocellular carcinoma	Lutein	Marigold (*Tagetes erecta* L.)	In vivo	-	Reduction in levels of alanine transaminase, aspartate transaminase, and alkaline phosphatase Increase in levels of glutathione Reduction in activity of c-glutamyl transpeptidase	[127]
Lung cancer Cervical cancer Breast cancer Hepatocellular carcinoma Cholangiocarcinoma	Extracts of *Chlorella* (Gallic acid and lutein) (EtOH)	*Chlorella* sp.	In vitro A549, Hela, MCF7, Huh7, CCA, KKU213A cells	400 μg mL^−1^	Inhibition of growth in all types of tumor cells considered Reduction in procaspase-3, -8, and -9 levels, increase in caspase activity, and decrease in anti-apoptosis protein Bcl-2, leading to cell death Reduction in levels of phosphorylated-AKT and phosphorylated-mTOR proteins, inhibiting AKT/mTOR survival signaling	[129]
Breast cancer Colorectal cancer Prostate cancer Skin melanoma	Extract of *Granulocystopsis* (MeOH)	*Granulocystopsis* sp.	In vitro HTB-22, HTB-38, HTB-81 and HTB-72 cells	IC50 for 48 h: 16.70 µg/mL 17.20 µg/mL 13.74 µg/mL 17.44 µg/mL	Differential reactions of cell lines to the extract, but viability decreases in all depending on treatment duration Favorable induction of apoptosis in skin melanoma and prostate cancer cell lines Increase in caspase 3 and 7 activity in all treated tumor cells	[131]
Head and neck cancer (HNC): Oral and pharyngeal cancer Laryngeal cancer	Carotenoids: bcryptoxanthin, lycopene, and lutein plus zeaxanthin	Fruit and vegetables	Epidemiological studies	-	Carotenoids exhibit antioxidant, antimutagenic, and immunoregulatory actions Inverse association observed between carotenoid intake and risk of head and neck cancer (HNC)	[137]
Bladder cancer (BC)	Carotenoidis: lutein, αcarotene, βcarotene, lycopene, βcryptoxanthin, and zeaxanthin	Fruit and vegetables	Epidemiological studies	-	Inverse association between total carotenoid intake and risk of BC	[138]
Renal cell carcinoma (RCC)	Carotenoids: αcarotene, βcarotene, lutein, zeaxanthin, and lycopene	Fruit and vegetables	Epidemiological studies	-	Inverse associations between total carotenoid intake and risk of RCC	[139]

## 4. Enhancement of Lutein Production in Microalgae

Although the lutein content in some terrestrial plants may be even higher than in microalgae, lutein production in microalgae can be enhanced by subjecting cell cultures to various types of stresses. The main conditions identified in the literature are summarized below and indicated in Table 2.

### 4.1. Metabolism

Microalgae exhibit diverse metabolic modes, including photoautotrophic, heterotrophic, mixotrophic, and photoheterotrophic modes according to their utilization of different sources of energy and carbon. Heterotrophic microalgae depend on organic carbon as the source of both energy and carbon, enabling them to proliferate in the absence of light. Photoautotrophic microalgae employ light as source of energy and inorganic carbon, such as CO_2_ or carbonate salts, as a source of carbon. Photoheterotrophic cultures utilize light energy and organic carbon, but they require light and cannot survive in darkness. Mixotrophic microalgae exhibit a versatile capacity to use both light energy and organic carbon as their energy sources, enabling the assimilation of both organic and inorganic carbon. Mixotrophic microalgae can conduct photosynthesis while also assimilating organic carbon. Importantly, the metabolic mode plays a pivotal role in influencing the ability of microalgae to accumulate lutein. For example, Yen et al. observed a preference for the autotrophic cultivation mode over mixotrophic cultivation mode in lutein production [154]. In a different study, Heo et al. demonstrated that, in *Parachlorella* sp. JD-076, autotrophic cultivation with 5% CO_2_ is more effective for lutein accumulation than mixotrophic culture, both in the presence of glucose and when glucose is present without CO_2_ [155]. Additionally, mixed strategies, involving an initial stage of mixotrophic cultivation followed by a second stage of autotrophic cultivation, were successfully implemented for lutein accumulation. This approach led to a lutein content of 11.22 ± 0.38 mg g^−1^ in *Chlorella sorokiniana* FZU60 [156].

### 4.2. Light

Light exerts a profound influence on lutein accumulation, with light quality, intensity, and supply strategies playing pivotal roles. The wavelength of light contributes to lutein accumulation, as evidenced by the fact that blue light enhances lutein content in *Chlamydomonas* sp. JSC4 [157]. In contrast, in *Scenedesmus obliquus* CWL-1, blue light does not increase lutein content but enhances lutein productivity [158].

Regarding light intensity, high lighting conditions generally lead to a decrease in lutein accumulation. For instance, Xie et al. demonstrated that increasing lighting conditions from 150 to 750 μmol m^−2^ s^−1^ resulted in a decrease in lutein content from 4.69 ± 0.08 to 3.30 ± 0.11 mg g^−1^ DW [159]. Similarly, other researchers observed that *Chlorella sorokiniana* Kh12 decreased lutein content when cultivated under stronger light intensities [160]. Indeed, exceptions to the general trends in light influence on lutein accumulation exist, as highlighted by Heo et al. They observed that *Parachlorella* sp. JD-076 increased lutein content up to 11.8 mg g^−1^ when exposed to an increased light intensity of 1000 μmol m^−2^ s^−1^, indicating a positive response to higher light levels [155]. Additionally, Ma et al. demonstrated a relationship between illumination intensity and lutein accumulation in *Chlamydomonas* sp., revealing an optimum intensity of 625 μmol m^−2^ s^−1^ for maximizing lutein accumulation before observing a decrease in content [161]. These exceptions highlight the complexity of the interplay between light conditions and lutein production in different microalgal species.

The method of light supply also plays a crucial role. Dineshkumar et al. observed that *Chlorella minutissima* accumulated 8.24 ± 0.12 mg g^−1^ and 7.96 ± 0.14 mg g^−1^ when linear and exponential light-feeding strategies were applied to the culture, outperforming the continuous lighting strategy with 6.37 ± 0.11 mg g^−1^ and leading to considerable energy savings [162].

### 4.3. Nitrogen

Nitrogen plays a crucial role in lutein accumulation, and the choice of nitrogen source significantly influences lutein concentration in microalgal cells. Shi et al. demonstrated that among nitrate, ammonium, and urea, urea proves to be the most effective nitrogen source for accumulating lutein in the heterotrophic culture of *C. protothecoides* [21]. In a different study, Ho et al. found that among Ca(NO_3_)_2_, (NH_4_)_2_SO_4_, and urea, a concentration of 8 mM Ca(NO_3_)_2_ was the most effective for lutein accumulation, resulting in 4.61 ± 0.11 mg g^−1^ in *Scenedesmus obliquus* FSP-3 grown in a photobioreactor [163]. In another case, the lutein production of *Chlorella sorokiniana* MB-1-M12 showed no significant differences for cultures grown with nitrate and ammonium, but a relatively lower level of lutein content was obtained when urea was used as a nitrogen source [164].

Regarding nitrogen concentration, maintaining nitrogen sufficiency is generally essential for achieving a high concentration of lutein. For instance, *Tetraselmis* sp. CTP4 accumulated up to 3.17 ± 0.18 mg g^−1^ DW when cultivated with a two-stage strategy in which, in the second stage, it was cultivated under nitrogen repletion (N-NO_3_), at 35 °C and 170 μmol m^−2^ s^−1^. [165]. Similarly, Ho et al. showed that in *S. obliquus* FSP-3, the content of lutein increased with the concentration of nitrogen in the medium, reaching 4.95 ± 0.22 mg g^−1^ with 24 mM of Ca(NO_3_)_2_ [163]. However, there seems to be an optimal concentration of nitrogen to optimize lutein content. Xie et al. demonstrated that the lutein content of *Desmodesmus* cultivated in a batch increased from 3.76 ± 0.23 mg g^−1^ DW to 4.97 ± 0.05 mg g^−1^ DW1 when nitrate concentration increased from 4.4 to 13.1 mM, but then decreased to 4.29 ± 0.13 mg g^−1^ DW when the nitrate concentration was 17.6 mM [159]. Furthermore, Xie et al. observed similar findings, with the content of lutein reaching a maximum of 5.56 mg g^−1^ in *Desmodesmus* sp. F51 when ammonium concentration was increased to 150 mg L^−1^, decreasing when nitrogen concentration further increased [166]. In their study on *Chlorella sorokiniana* FZU60, Xie et al. demonstrated that a higher concentration of NaNO_3_ (1 g L^−1^) resulted in the optimal lutein accumulation of 9.65 ± 0.25 mg g^−1^ [167]. Similar results were found by other authors [168]. In contrast, Shi et al. observed that nitrogen limitation enhanced lutein production by *Chlorella protothecoides* in heterotrophic fed-batch cultures of approx. 0.27 mg g^−1^ in the N-limited culture [169]. Despite reports indicating that nitrogen limitation can boost the synthesis of secondary carotenoids in certain algae species, lutein, typically considered a primary carotenoid, generally does not experience increased production under nitrogen deprivation. Nevertheless, in the case of heterotrophic growth, where algae are grown without relying on light, carotenoids might not serve the same function as they do in phototrophic mode. This distinction in function may explain the observed differences in regulation under nitrogen limitation in heterotrophic conditions.

### 4.4. Temperature

Temperature significantly influences lutein accumulation, generally with higher temperatures promoting increased lutein synthesis. For instance, *Tetraselmis* sp. CTP4 demonstrated enhanced lutein accumulation, reaching up to 2.15 ± 0.25 mg g^−1^ DW at a salinity of 35‰ and 30 °C, compared to 1.64 mg g^−1^ DW under standard conditions of 20 °C and a salinity of 35‰ [165]. Similarly, in *Chlorella protothecoides*, an increase in temperature from 24 to 32 °C led to a 25% rise in lutein content [169]. It was also reported that lutein concentration increased with temperature from 26 to 32 °C in *Chlorella sorokiniana* [170]. However, there is an optimal temperature range for lutein accumulation [156,160]. In contrast, *Chlamydomonas* sp. exhibited higher lutein accumulation when cultivated at 20–25 °C compared to higher temperatures [157]. The accumulation of lutein at low temperatures can be attributed to its role in regulating membrane fluidity. Carotenoids, including lutein, are proposed to play a crucial role as a biological adaptation to low temperatures. Specifically, they contribute to the regulation of intracellular thylakoid membrane fluidity, serving as a mechanism to counteract the negative effects associated with lower temperatures [157]. 

### 4.5. Organic Carbon Source

The choice of organic carbon source significantly impacts lutein production. For example, in the extremophile *Chamydomonas acidophila*, amongst several other carbon sources, in the presence of CO_2_, starch, urea, or glucose were the best carbon source, leading to an accumulation of 10 mg g^−1^ DW [171]. Another microalga, *Chlorella sorokiniana* MB-1-M12, showed a preference for acetate over glucose in lutein accumulation [164]. The concentration of the organic carbon source in the growth medium plays a role in lutein accumulation. In the growth of *C. protothecoides*, optimal concentrations of glucose were determined to be 10 g L^−1^, leading to a concentration of 4.38 mg g^−1^ DW after 92 h of cultivation, which increased to 4.44 mg g^−1^ DW after 238 h of cultivation [172]. Conversely, in the microalga *C. sorokiniana* Mb-1 grown in mixotrophic mode, lower acetate concentrations resulted in better lutein accumulation. When the acetate concentration was 1 g L^−1^, the lutein content reached 4.60 mg g^−1^ [168]. Likewise, Xie et al. found that *Chlorella sorokiniana* FZU60 accumulated more lutein when 1 g L^−1^ of sodium acetate was used; however, the lutein accumulation gradually decreased with an increase in acetate concentration [167].

### 4.6. Inorganic Carbon Source

The inorganic carbon source also plays a crucial role in lutein accumulation. In *Desmodesmus* sp. F51, the best lutein accumulation was achieved under a 2.5% CO_2_ supply, surpassing the addition of NaHCO_3_ or Na_2_CO_3_. When both CO_2_ supply and NaHCO_3_ were used, the concentration reached 3.98 ± 0.13 mg g^−1^ [166]. Additionally, CO_2_ concentration in autotrophic mode influences lutein accumulation, as demonstrated by Molino et al. in *Scenedesmus almeriensis*. This microalga accumulated the highest amount of lutein (5.71 mg g^−1^) under the highest tested CO_2_ concentration [173].

### 4.7. Abiotic Stress

Various abiotic stresses have been explored to understand their impact on lutein accumulation. Oxidative stress, for instance, demonstrated the ability to promote lutein formation and enhance lutein production in heterotrophic *Chlorella protothecoides* [174]. The cells likely increased lutein production to counteract ROS present in the growth medium. Specifically, the presence of 0.01 mmol L^−1^ H_2_O_2_ and 0.5 mmol L^−1^ NaClO resulted in an increase in lutein content from 1.75 to 1.98 mg g^−1^ DW. Salinity also influences lutein accumulation, generally leading to a decrease [160,170]. However, in the case of *Coccomyxa onubensis*, Bermejo et al. found that lutein content increased with salinity, reaching 7.80 mg g^−1^ with 500 mM of NaCl, although lutein productivity was optimized at 100 mM NaCl [175]. In the same research, they observed that illumination with photosynthetically active radiation (PAR)+UVA light increases lutein content to 7.07 mg g^−1^. Researchers have also evaluated pH effectiveness in accumulating lutein. In *Chlorella sorokiniana* MB-1-M12, it has been shown that the optimal pH for lutein accumulation was 7.5 [164].

### 4.8. Growth Strategy

Fed-batch conditions have proven to be effective in enhancing lutein content in microalgal cells. For instance, Xie et al. demonstrated that employing a fed-batch strategy with a continuous supply of 2.2 mM nitrate after the initial nitrate concentration was depleted maximized the lutein concentration in *Desmodesmus* sp. F51 to 5.05 ± 0.20 mg g^−1^ [159]. Other researchers observed similar findings when cultivating *Chlorella protothecoides* in fed-batch mode [169]. The effectiveness of the fed-batch strategy in inducing lutein accumulation was also confirmed by Ma et al. [176], by Xie et al. [167], and by Chen et al. [158]. In another case, a novel two-stage process integrating fed-batch and semi-batch modes was shown to be effective in promoting lutein accumulation [164].

**Table 2:** Main culturing conditions used for lutein production optimization by microalgae.

Microorganism	Culture Conditions	Metabolic Mode	Enhancing Condition Applied	Lutein Content	Comment	Ref.
*Tetraselmis* sp. *CTP4*	Batch cultivation in 5-L reactors with modified algal medium at 100 μmol m^−2^ s^−1^	Photoautotrophic	Temperature	2.15 ± 0.25 mg g^−1^	Lutein content demonstrates an increase with rising temperature, reaching optimal levels at 30 °C	[165]
*Tetraselmis* sp. *CTP4*	First step: batch cultivation in 5 L reactors with modified algal medium	Photoautotrophic	Combination of several strategies	3.17 ± 0.18 mg g^−1^	Effective enhancement of lutein content achieved through a two-stage cultivation process involving nitrogen depletion, controlled temperature, and light optimization	[165]
*Chlorella protothecoides*	Batch cultivation in 3.7 L fermenter with modified basal medium	Heterotrophic	Glucose addition	4.44 mg g^−1^	Optimal glucose concentrations were found to be 10 g L^−1^ and 40 g L^−1^	[172]
*Chlorella protothecoides*	Batch cultivation in 3.7 L fermenter with modified basal medium with 40 g L^−1^ of glucose	Heterotrophic	Nitrogen source	4.58 mg g^−1^	Urea identified as the most effective nitrogen source for enhancing lutein content	[21]
*Chlorella protothecoides*	Batch cultivation in modified basal medium in Erlenmeyer flasks (250-mL)	Heterotrophic	Oxidative stress	1.98 mg g^−1^	Increased lutein accumulation observed in the presence of 0.01 mmol L-1 H_2_O_2_ and 0.5 mmol L^−1^ NaClO	[174]
*Chlamydomonas acidophila*	Batch cultivation in 1 L reactor at 25 °C	Mixotrophic	Source of carbon	10 mg g^−1^	In the presence of CO_2_, starch, urea, or glucose were the best carbon source	[171]
*Desmodesmus* sp. *F51*	Batch cultivation in photobioreactor at 150 μmol m^−2^ s^−1^, temperature at 35 °C	Photoautotrophic	Nitrogen	4.97 ± 0.05 mg g^−1^	Optimal lutein accumulation achieved at 13.2 mM of N-nitrate	[159]
*Desmodesmus* sp. *F51*	Batch cultivation in photobioreactor (PBR) at 150 μmol m^−2^ s^−1^, temperature at 35 °C; nitrate concentration, 8.8 mM	Photoautotrophic	Light intensity	4.69 ± 0.08 mg g^−1^	Increasing light intensity leads to a decrease in lutein content	[159]
*Desmodesmus* sp. *F51*	Fed-batch photobioreactor at 600 μmol m^−2^ s^−1^ at 35 °C temperature, initial nitrate concentration, 8.8 mM	Photoautotrophic	Growth strategy	5.05 ± 0.20 mg g^−1^	Increased lutein content observed in fed-batch cultivation with a 2.2 mM nitrate feeding concentration	[159]
*Chlorella protothecoides*	Fed-batch growth; batch condition with 40 g L^−1^ of glucose; fed-batch condition limited in nitrogen	Heterotrophic	Combination of several strategies	5.35 mg g^−1^	Effective enhancement of lutein content achieved through a three-step cultivation process involving growth strategy, nitrogen depletion, and temperature control	[169]
*S. obliquus FSP-3*	Batch growth in photobioreactor, 24 mM Ca(NO_3_)_2_	Photoautotrophic	Nitrogen concentration	4.95 ± 0.22 mg g^−1^	Lutein content shows a positive correlation with rising nitrogen concentration	[163]
*Chlorella minutissima MCC-27*	Batch cultivation in 2 L airlift photobioreactor with BBM	Photoautotrophic	Light supply	8.24 ± 0.12 mg g^−1^	Light supply strategy has a role on lutein accumulation	[162]
*Desmodesmus* sp. *F51*	Batch cultivation in 1 L glass photobioreactor with modified Bristol’s medium at 150 μmol m^−2^ s^−1^	Photoautotrophic	Carbon and nitrogen sources	5.56 mg g^−1^	Optimal lutein accumulation achieved at 150 mg L^−1^ of N-ammonium	[166]
*Chlorella sorokiniana Mb-1*	Batch cultivation in 1 L glass photobioreactor with modified Bristol’s medium at 150 μmol m^−2^ s^−1^ with 1.83 g L^−1^ of nitrate	Mixotrophic	Organic carbon concentration	4.6 mg g^−1^	Increased lutein content observed at the lowest tested concentrations of acetate (4.88 g L^−1^)	[168]
*Chlamydomonas* sp. *JSC4*	Batch cultivation in photobioreactor with modified Bold 3 N medium, sea salt, and initial nitrate-N concentrations adjusted to 2% and 1000 mg L^−1^	Photoautotrophic	Light quality	3.10 mg g^−1^	Blue light increases lutein content	[157]
*Chlamydomonas* sp. *JSC4*	Batch cultivation in photobioreactor with modified Bold 3 N medium/two-stage cultivation	Photoautotrophic	Combination of several strategies	4.24 ± 0.22 mg g^−1^	Efficient enhancement of lutein content achieved through two-stage cultivation involving control of light quality and temperature	[157]
*Chlorella sorokiniana C16*	Batch cultivation on Tris acetate phosphate (TAP) media	Mixotrophic	Temperature	17.4 mg g^−1^	Lutein content exhibits a positive trend, reaching its peak with increasing temperature up to 32 °C	[170]
*Chlorella sorokiniana FZU60*	Batch cultivation in BG11 medium at 33 °C; light intensity, 150 µmol m^−2^ s^−1^, initial NaNO3 concentration of 1 g L^−1^	Mixotrophic	Organic carbon concentration	9.65 ± 0.25 mg g^−1^	Optimal lutein accumulation achieved at 1 g L^−1^ of sodium acetate	[167]
*Chlorella sorokiniana FZU60*	Batch cultivation in BG11 medium; light intensity, 150 µmol m^−2^ s^−1^, 0.75 g L^−1^ sodium nitrate, and 1 g L^−1^ sodium acetate	Mixotrophic	Temperature	9.81 ± 0.49 mg g^−1^	Peak lutein content achieved at an optimal temperature of 33 °C	[156]
*Chlorella sorokiniana Kh12*	Batch cultivation on Tris acetate phosphate (TAP) media	Mixotrophic	Temperature	17.25 mg g^−1^	Peak lutein content achieved at an optimal temperature of 30 °C	[160]
*Scenedesmus almeriensis*	Batch cultivation in vertical bubble column photobioreactor in modified Mann & Myers medium	Autotrophic	CO_2_ concentration	5.71 mg g^−1^	Optimal lutein accumulation observed at the highest CO_2_ concentration tested (3% *v*/*v*)	[173]
*Parachlorella* sp. *JD-076*	Batch cultivation in tubular-type photobioreactor in BG-11 medium at 35 °C	Autotrophic	Light intensity	11.8 mg g^−1^	Lutein content shows a positive correlation with rising light intensities	[155]
*Coccomyxa onubensis*	Batch cultivation in 2L Erlenmeyer flasks 140 µmol m^−2^ s^−1^ at 26 °C	Autotrophic	Salt	7.80 mg g^−1^	Enhanced lutein content observed under UVA + PAR illumination	[175]
*Coccomyxa onubensis*	Batch cultivation in 2L Erlenmeyer flasks 140 µmol m^−2^ s^−1^ at 26 °C	Autotrophic	UVA light	7.07 mg g^−1^	Lutein content demonstrated a positive correlation with rising salinity levels	[175]
*Chlorella sorokiniana MB-1-M12*	Batch cultivation in 5L fermenter with BG-11 medium at 27 ± 1 °C	Heterotrophic	Two-stage process	5.88 mg g^−1^	The described two-stage process integrating fed-batch and semi-batch modes induced lutein accumulation	[164]
*Scenedesmus obliquus CWL-1*	Batch cultivation in 7 L stirred tank reactor with 4.5 g L^−1^ of calcium nitrate	Mixotrophic	Combination of several strategies	2.45 mg g^−1^	Photoperiod and wavelength adjustment effective in inducing lutein	[158]

## 5. Pilot-Scale Systems for Lutein Production 

Various attempts have been made to produce lutein on a pilot scale. In an early effort, Moulton et al. cultivated *Dunaliella viridis* at pilot scale to produce various carotenoids. Laboratory experiments showed robust growth of *D. viridis*, achieving high levels of mixed carotenoids (approximately 13 mg L ^−1^ carotenoid). However, outdoor pond experiments yielded less promising results [177]. Del Campo et al. [23] explored an alternative approach using a closed tubular reactor made of methyl polymethacrylate, equipped with an airlift system for cell culture recirculation and an external horizontal loop of tubes (90 m long, 2.4 cm inner diameter, and 2.2 m^2^ surface). They employed *Muriellopsis* sp. in semi-continuous mode, investigating the impact of dilution rate, mixing, and daily solar cycles on lutein and biomass productivity. Optimal productivity values, for both lutein (about 180 mg m−2 per day) and biomass (about 40 g m^−2^ per day), were achieved in May and July, with varying optimal dilution rates [23]. 

Jeon et al., after optimizing the medium recipe to enhance lutein productivity, validated the process on a larger scale of 25,000 L and 240,000 L. The lutein concentrations obtained at these scales (260.55 ± 3.23 mg L^−1^ and 263.13 ± 2.72 mg L^−1^, respectively) were comparable to the laboratory scale of 252.75 ± 12.92 mg L^−1^ [178].

McClure et al. identified optimal conditions for lutein production from *Chlorella vulgaris* in a 5 L photobioreactor, later validating the process at a 50 L scale. They achieved maximized lutein productivity (1.6 mg L^−1^ day^−1^) using specific parameters and demonstrated consistent system performance in semi-continuous operation for 32 days, maintaining high lutein concentrations (15–20 mg L^−1^) [179].

In a different study, Xie et al. evaluated the potential of *Chlorella sorokiniana* FZU60 for lutein production in a 50 L column photobioreactor using a two-stage strategy. Lutein content, production, and productivity reached 9.51 mg g^−1^, 33.55 mg L^−1^, and 4.67 mg L^−1^ d^−1^, respectively [180]. In another case, *Coccomyxa onubensis*, cultivated in an 800 L vertical tubular photobioreactor outdoors, demonstrated stable biomass production for at least one month, with a lutein content of 10 mg g^−1^ and a maximal lutein productivity of 1.42 mg L^−1^ d^−1^ [181]. The authors also cultivated the same microalga indoors, in two plastic bags of 400 L each. A maximal lutein concentration of 9.7 mg g^−1^ was obtained, leading to a maximal lutein productivity of 0.9 mg L^−1^ d^−1^.

More recently, Cavieres et al. integrated microalgal cultivation with wastewater treatment for carotenoid production. *Muriellopsis* sp. in an 800 L raceway removed 84% of nitrogen, 93% of phosphorus, and other chemical compounds within 4 days. Biomass productivity reached 104.25 mg·L^–1^·day^–1^, with 51% protein and a pigment content of 0.6% carotenoid, including 0.3% lutein [182]. Lastly, *Chlorella sorokiniana* TH01, cultivated in a 90 L flat-plate photobioreactor indoors, achieved the highest biomass and lutein productivity of 284–469 mg L^−1^ d^−1^ and 2.57–4.57 mg L^−1^ d^−1^, respectively [183]. 

The existing literature provides ample evidence supporting the technical feasibility of mass-producing lutein through microalgal cultivation. Over the years, various technical challenges have been addressed, indicating the viability of the process. To comprehensively assess the economic feasibility, additional evidence on technoeconomic aspects is essential. This will contribute to a more thorough understanding of the economic viability of the lutein production process.

## 6. Genetic Engineering Production

Many researchers have also tried to increase lutein production by metabolic engineering approaches. In the last few years, many attempts have been made to obtain encouraging yields. Chemical mutagenesis (such as with N-methyl-N′-nitro-N-nitrosoguanidine and ethyl methane sulfonate) as well as targeted genetic engineering of specific genes have been applied to the engineering of microalgal biosynthetic pathways for lutein production [184]. Huang et al., for instance, studied a mutant of *Chlorella zofingiensis* (CZ-bkt1) obtained by chemical mutagen (N-methyl-N’-nitro-N73 nitrosoguanidine) exposure. In addition, they cultivated it under different conditions and obtained the highest lutein production in high light irradiation and nitrogen deficiency (13.81 ± 1.23 mg g^−1^) and feeding with glucose culturing conditions (33.97 ± 2.61 mg L^−1^) [185]. *Chlorella sorokiniana* was also modified by random mutagenesis with N-methyl-N′-nitro-nitrosoguanidine (MNNG), obtaining a mutant MR-16 exhibiting 2.0-fold higher volumetric lutein content (42.0 mg L^−1^) and mutants DMR-5 and DMR-8 with lutein cellular content of 7.0 mg g^−1^ dry weight [186].

Regarding genetic engineering, transformation was generally performed by electroporation, shaking of cells with glass beads, and particle gun bombardment (biolistic method). The most studied genes were phytoene synthase (psy), phytoene desaturase (pds), and phytoene-β-carotene synthase (pbs). Cordero et al. studied the phytoene synthase gene, involved in the first step of carotenoid synthesis, from the green microalga *Chlorella zofingiensis*. The phytoene synthase gene was expressed in *Chlamydomonas reinhardtii* by nuclear transformation, resulting in gene overexpression and an increase of lutein production 2.2-fold higher with respect to untransformed cells [187]. Similarly, Liu et al. studied the gene phytoene desaturase from the green microalga *Chlamydomonas reinhardtii*. The authors applied a single amino acid substitution, L505F, and showed that this modification induced an increase of 29% of its activity and increased production of carotenoids [188]. In another similar study, Liu at al. nuclear-transformed *Chlorella zofingiensis* with a mutant version of phytoene desaturase gene (one amino acid substitution). This modification induced an increased production of carotenoids as well (total carotenoids 32%) compared to the control strain [189]. Recently, Rathod and co-workers inserted the gene coding for phytoene-β-carotene synthase from the red yeast *Xanthophyllomyces dendrorhous* in *Chlamydomonas reinhardtii*, by nuclear transformation. They obtained a species with the bifunctional enzyme with both phytoene synthase and lycopene cyclisation activities and obtained a 60% increase in lutein production under low light conditions [190].

Takemura et al. in 2021 applied heterologous expression by using as host *Escherichia coli*. In particular, they included, in the *E. coli* JM101 (DE3) genome, various genes involved in the lutein biosynthetic pathway, such as lycopene β-cyclase, lycopene ε-cyclase, and cytochrome P450 97C. They introduced in *E. coli* several plasmids containing some of the multiple genes and compared lutein productivity by obtaining 11 mg L^−1^ [191]. In the same year, Bian et al. tried to obtain good yields by heterologous expression in yeast and in particular in *Saccharomyces cerevisae*. During their experiments, they found as limiting step a competition of the cyclases for lycopene, forming β-carotene, and solved this problem by a temperature-responsive expression redirecting to α-carotene synthesis. At the end, they obtained 438 μg g^−1^ dry cells [192].

## 7. Conclusions

Overall, the information reported in this review shows that microalgae can be considered the main source of lutein, and carotenoids in general, in the marine environment [193]. Their use as lutein producers is advantageous compared to terrestrial plants because they are characterized by higher growth rates, combined with high photosynthetic efficiency and a great potential for carbon mitigation. Actually, the commercial supply of lutein is characterized by the bright yellow petals of the marigold flowers of the genus *Tagetes*, while chemical synthesis allows one to obtain very low yields [13]. Hence, novel eco-sustainable sources like microalgae can be a valid alternative, thanks also to the possibility of cultivating them in a controlled manner in photobioreactors.

In addition, nowadays, researchers are looking for new extraction methods that are environmentally sustainable. In fact, to overcome the high dosage of solvents and organic bases that are required in conventional methods of lutein extraction, researchers are focusing on the optimization of the extraction process, trying to limit environmental impacts [33]. Overall, it is always important to consider the environmental impact during the extraction process of natural substances, and therefore the suggestion is to try to use green solvents, such as vegetable oils, to perform extractions at low temperatures and/or with ultrasounds by using low energy consumption and reducing the extraction timing. 

## Data Availability

Not applicable.
